# Supplementary material for: Intermittent Fasting, Dietary Modifications, and Exercise for the Control of Gestational Diabetes and Maternal Mood Dysregulation: A Review and a Case Report
Source: Int J Environ Res Public Health. 2020 Dec 15;17(24):9379. doi: 10.3390/ijerph17249379 (PMC7765295; doi:10.3390/ijerph17249379)
Supplement: Supplementary file 1 [file ijerph-17-09379-s001.pdf]

Supplementary File 1. Patient's narrative of her psychological distress in relevance to her GDM experience

I think that my sleep was awful. I had to wake up several times to go to the toilet, and when I went back to bed, it took me a long time to fall asleep. While waiting for sleep to strike me, I had thoughts that I may feel full and need to go to the toilet again soon, which would really make me feel uncomfortable. Even the short time I slept was full of vague dreams, and when I got up, I had headache and I felt down and blue may be for one or two hours until I go out for a walk to feel that I am back to myself again. I used to measure my blood pressure every day and it was always normal. I thought that my poor night sleep would be the cause of the headache.

Preparing breakfast for the kids, and helping them change their closes, pack their lunch box and taking them to the nursery became a big load. Though I could do all my daily chores, I felt things were getting harder and that I need some relief or help.

I had 2 cesarean section operations before, and that one would also be born through cesarean operation. I know a GDM women who had sharp and severe clotting in her blood when she went through this operation. She died and her baby developed jaundice and was kept in the neonatal ICU for a long time. I worried that I may be another scenario of that woman. I felt that I may not be able to make it this time and kept thinking what if my wound does not recover, what if I become diabetic for the end of my life, what if my baby develops something wrong in his internal organs or his brain structure gets damaged as a result of my high glucose. I felt that even my obstetrician was worried, and she wanted me to be admitted to hospital though my sugar was not that high. She kept warning me that although she checked fetal organs and that they look good, some anomalies may have not been detected. She also kept emphasizing that blood transfusion may be necessary at labor time. All these stuffs made me feel tense. GDM was a really terrible experience for me.

When I started to monitor my blood glucose before and after meal, I noticed that over a short time my 2 hours glucose was increasing to approach or even exceed 120. Meal time became a great stress for me. I felt that eating means that I have a great mission to do…. reducing my glucose level otherwise I will be killing my baby when I eat. I felt that it was not enough to eat food of a good quality. Once I ate only lettuce and 2 table spoons on yoghurt, and my 2 hours glucose increased to above 120. I felt that my blood sugar may rise even when I drink water without eating anything. I had to exercise harder to prevent it. Even when I exercised, my tension did not allow me to control it. One night my glucose was above 130 two hours after dinner. So, I went for a walk again, and I measured it again. Shockingly, it increased to above 160. I had a feeling that it was getting out of my hand. My husband checked some GDM forums on the internet that night, and he assured me that I am not alone in this and some people have similar complaints. I just kept focused on controlling my glucose all the time, and I felt that I could not do it anymore. Just thinking about drained a lot of my psychic energy. I told the doctor that maybe I need to take insulin or another medicine, but he said that my blood glucose is under control and if it gets worse, he would prescribe me insulin. I guess he could not realize how hard I tried to keep it under control. So, I just had to go and suffer it all by myself. Finally, I managed to watch some experts on YouTube, including diabetic patients who explained how they managed to control their blood glucose through fasting. So, I decided to have 2 meals and keep fasting during day time so that when I see the food I tell myself that I am not allowed to eat.
